# Oxidation is a potentially significant methane sink in land-terminating glacial runoff

**DOI:** 10.1038/s41598-024-73041-3

**Published:** 2024-10-08

**Authors:** Kristin E. Strock, Rachel B. Krewson, Nicole M. Hayes, Bridget R. Deemer

**Affiliations:** 1 Environmental Science Department, Dickinson College, Carlisle, PA 17013 USA; 2 Biology Department, University of Wisconsin Stout, Menomonie, WI 54751 USA; 3 US Geological Survey, Southwest Biological Science Center, Flagstaff, AZ 86001 USA

**Keywords:** Methane, Glaciers, Lakes, Rivers, Greenhouse gas emissions, Limnology, Carbon cycle, Limnology

## Abstract

Globally, aquatic ecosystems are one of the largest but most uncertain sources of methane, a potent greenhouse gas. It is unclear how climate change will affect methane emissions, but recent work suggests that glacial systems, which are melting faster with climate change, may be an important source of methane to the atmosphere. Currently, studies quantifying glacial emissions are limited in number, and the role of methanotrophy, or microbial methane oxidizers, in reducing atmospheric emissions from source and receiving waters is not well known. Here we discuss three potential sites for methane oxidation that could mitigate emissions from glaciers into the atmosphere: under ice oxidation, oxidation within proglacial lakes, and oxidation within melt rivers. The research presented here increases the number of glacial sites with methane concentration data and is one of only a few studies to quantify the net microbial activity of methane production and oxidation in two types of land-terminating glacial runoff (lake and river). We find that oxidation in a glacial river may reduce atmospheric methane emissions from glacial melt by as much as 53%. Incorporating methane oxidation in estimates of glacial methane emissions may significantly reduce the estimated magnitude of this source in budgeting exercises.

## Introduction

Climate change is altering arctic environments, including sea ice thinning^1^, permafrost melting^2^, and earlier melting of ice in lakes and rivers^3^. Glacial melt is also accelerating with rapid loss of ice volume reported in 260 small glaciers across the globe^4^. Cross-ecosystem transport of water, geologic material, carbon and nutrients from ice sheets subsidizes terrestrial and aquatic ecosystems in paraglacial landscapes^5^ and transport could accelerate with increased melt.

While glacier systems are not currently included in global aquatic methane budgets^6^, existing studies suggest that they may be an important global source. The limited studies that have measured methane concentrations in glacial melt suggest that sub-glacial environments are conducive to biological and thermogenic methane production, and that methane accumulates under ice sheets and flushes out during melting events^7–11^. Methane emissions from glacial melt have only been reported from four studies (Sólheimajökull Glacier in southern Iceland^7^, Leverett Glacier on the Greenland Ice Sheet^8^, 3 alpine glaciers in Yukon, Canada^11^, and 78 land terminating glaciers across central Svalbard^10^) but anoxic conditions and microbial communities with methanogenic potential are present in subglacial environments, suggesting the production and storage of methane beneath glaciers could be more significant and extensive than previously anticipated^12–15^. Lamarche-Gagnon et al.^8^ estimated that ~ 6.3 Mg of methane were transported from under the Leverett Glacier during the 2015 melt season. Burns et al.^7^ estimated methane flux away from Sólheimajökull Glacier to be 48 Mg d^− 1^ at the glacial bed and 41 Mg d^− 1^ released to the atmosphere from the river during the summer melt season (60 days). Kleber et al.^10^ estimated that 2,310 Mg of methane are released from proglacial springs in the Svalbard archipelago each year.

Rough upscaling of these rates based on the relative surface area of these glaciers results in global glacier melt fluxes of 0.21 Tg CH_4_ yr^− 1^ (based on the Leverett Glacier size and flux), 0.25 Tg CH_4_ yr^− 1^ (based on the surface area and flux from Svalbard glaciers), and 185 Tg CH_4_ yr^− 1^ (based on the Sólheimajökull Glacier size and flux, see methods for more detail on upscaling). On the low end, this flux is insignificant to global budgets, on the high end; however, this flux comprises ~ 20 to 34% of annual global emissions (with global emission source estimates ranging from 538 to 884 Tg CH_4_ yr^− 1^)^16,17^. Order of magnitude differences in methanogenic potential of subglacial meltwater in land-terminating glaciers may occur due to differences in substrate type, among other factors^15^. The magnitude of glacially derived methane that reaches the atmosphere via receiving waters depends both on the methane delivered from the glacier and the degree of microbial oxidation in the receiving systems. Thus, understanding the rates and controls on methane flux is key to understanding how receiving waters may mitigate the degree to which glacial methane reaches the atmosphere.

Microbial oxidation is a sink for methane from ocean, lake, and wetland ecosystems, and can reduce atmospheric emissions by as much as 99% via both aerobic and anaerobic pathways^18–20^. It has been argued that anoxia in subglacial environments generally limits methane oxidation^15^. Still, observations of high microbial methane oxidation under the West Antarctic Ice Sheet^21^ and in subglacial sediments^7^ suggest that under-ice oxidation significantly reduces the methane escaping from glacial systems. Studies reporting high methane emissions from glaciers have acknowledged this under-ice methane oxidation^7,8^, but have discounted the role of methane oxidation in the paraglacial floodplain and receiving waters. Although the role of methane oxidation in paraglacial rivers is not well constrained, high rates of microbial methane oxidation have been reported in Greenland marginal rivers^9^ and in Chalk River sediments in the United Kingdom^22^. In the Greenlandic sites, aerobic incubations of river water resulted in the consumption of 98% of ambient methane over a 30-day period, suggesting oxidation has the potential to limit methane emissions at this site, but the ultimate magnitude of methane reduction depends on the water residence time. It follows that microbial communities poised in oxygenated drainage channels could serve as significant methane sinks more broadly.

**Fig. 1:**
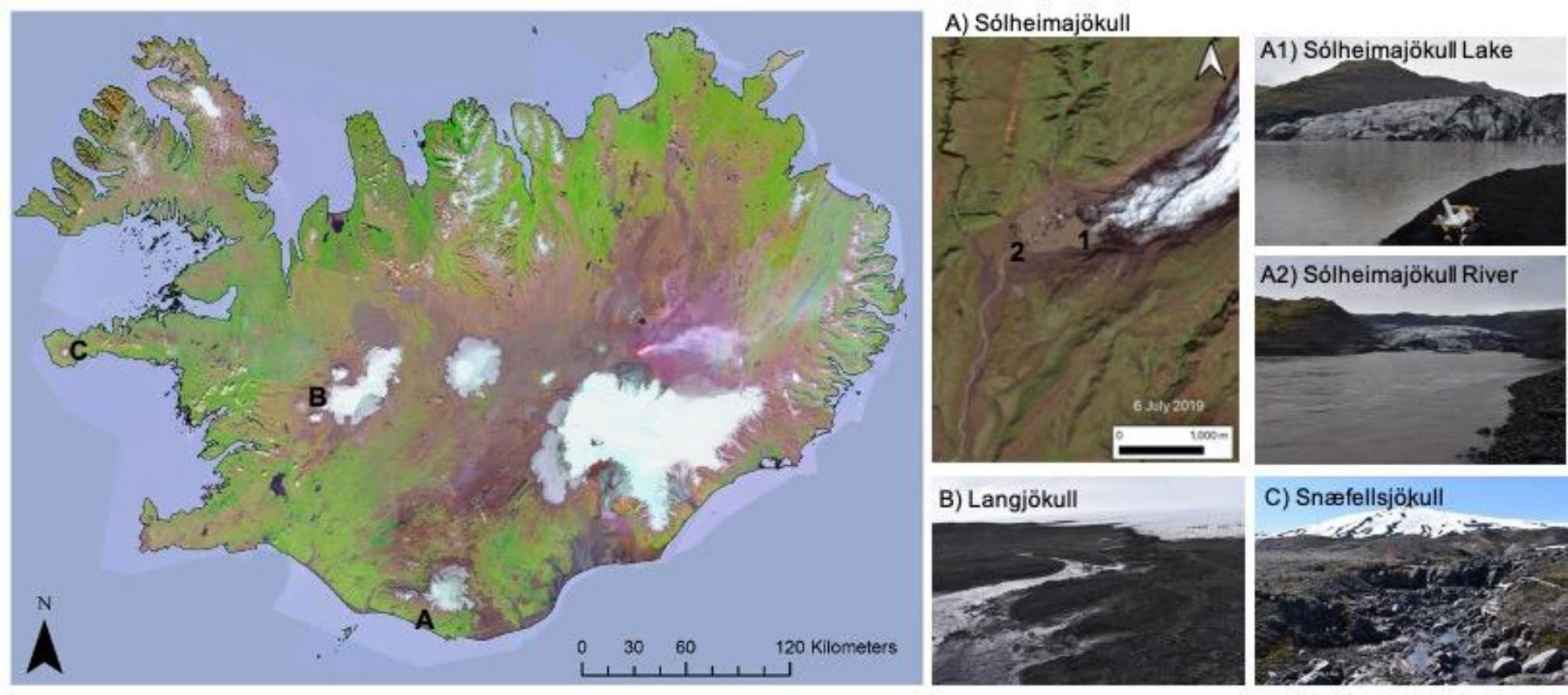
Sites in southern and western Iceland. Methane concentration measurements were taken within 50 m of three land-terminating glaciers (**A1** and **A2**, **B** and **C**) and in-situ methane oxidation experiments were completed in a paraglacial lake (**A1**) and river (**A2**). This map includes Landsat-8 images courtesy of the U.S. Geological Survey (https://www.usgs.gov/core-science-systems/nli/landsat/landsat-8) and was produced using Arc GIS Pro software version 3.1.2. The Landsat8 mosaic of Iceland was based on imagery from 2013 and 2014 and was produced by the National Land Survey of Iceland (http://gis.lmi.is/geoserver/wms? layername: Landsat8_mosaic 2014_5000dpi1 accessible via the Land Information System). Photos taken by Rachel Krewson.

In the present study, we sampled proglacial meltwaters of three Iceland glaciers that are experiencing glacial retreat^23^. To quantify methane concentrations, duplicate surface water samples were collected from the base of three Icelandic glaciers (Fig. 1;<50 m from the glacial margin) during high melt (19–21 June 2019). Diffusive methane fluxes were estimated using measured methane concentrations and estimates of gas transfer rates (based on energy dissipation rates in the paraglacial river and wind speed over the paraglacial lake). At one glacier, we also conducted net methane oxidation assays in paraglacial lake and river water to ask whether oxygenated paraglacial sediments can serve as sites of net microbial oxidation (Fig. 1). For this, we set up 6 bottles per site, adding 5mL of lake or river sediment to 65mL of overlying water. Three bottles were treated as initial concentrations, and three bottles were incubated in situ for 24 h before being sampled for a final concentration. The resulting rates represent the balance between methane production and oxidation in the system, with declines in methane concentration indicating net methane oxidation. Finally, we aim to place the oxidation rates we measured in the context of overall methane emissions originating from subglacial environments.

## Results and discussion

### Concentrations and diffusive fluxes

Both the paraglacial lake and the river were supersaturated, with 0.34 and 0.53 μM excess CH_4_ respectively. We estimate a gas transfer velocity (k600) of 0.3 and 205.4 m d ^− 1^ for the lake and river respectively, resulting in estimated diffusive fluxes of 0.05 and 63 µmol CH_4_ m^− 2^ d^− 1^.

## Oxidation across the paraglacial landscape

**Fig. 2:**
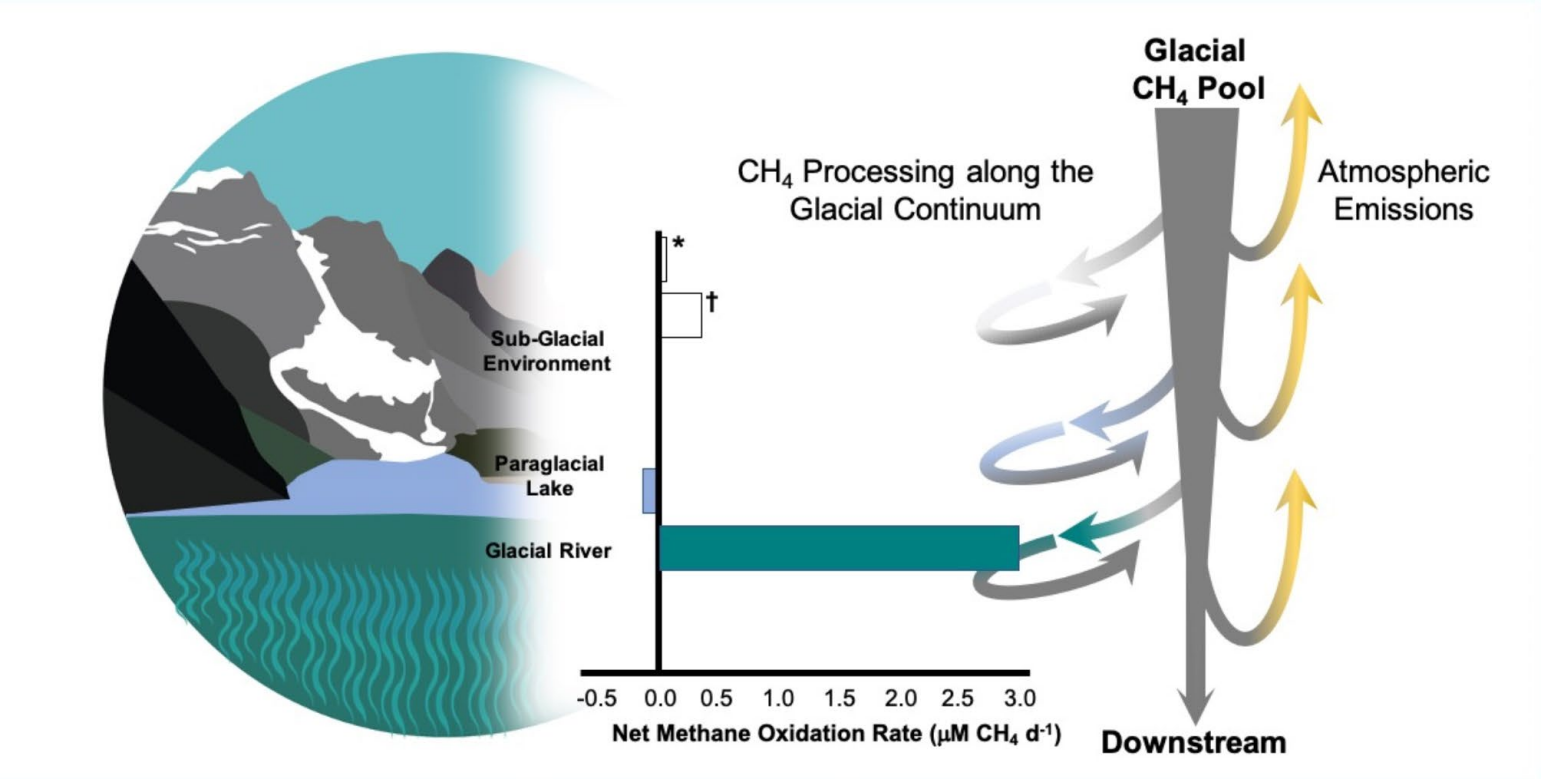
Three potential sites for methane oxidation that could mitigate atmospheric methane from glacial environments: under ice oxidation in sub-glacial environments, oxidation within paraglacial lakes (a common feature in glacial landscapes), and oxidation within glacial rivers that transport geological material from the glacial surface. Bar plot shows existing methane oxidation rates for sub-glacial environments (*Michaud et al.^21^ reported > 99% of Antarctic sub-ice sheet methane consumed via oxidation; ^†^Dieser et al.^9^ reported > 98% of methane was consumed from sub-glacial water over 30 days under aerobic conditions), and the net methane oxidation rates from paraglacial lakes and glacial rivers we report here. We review evidence for methane oxidation in all of these environments and report some of the first methane oxidation values for paraglacial lakes and rivers in the literature.

**Fig. 3:**
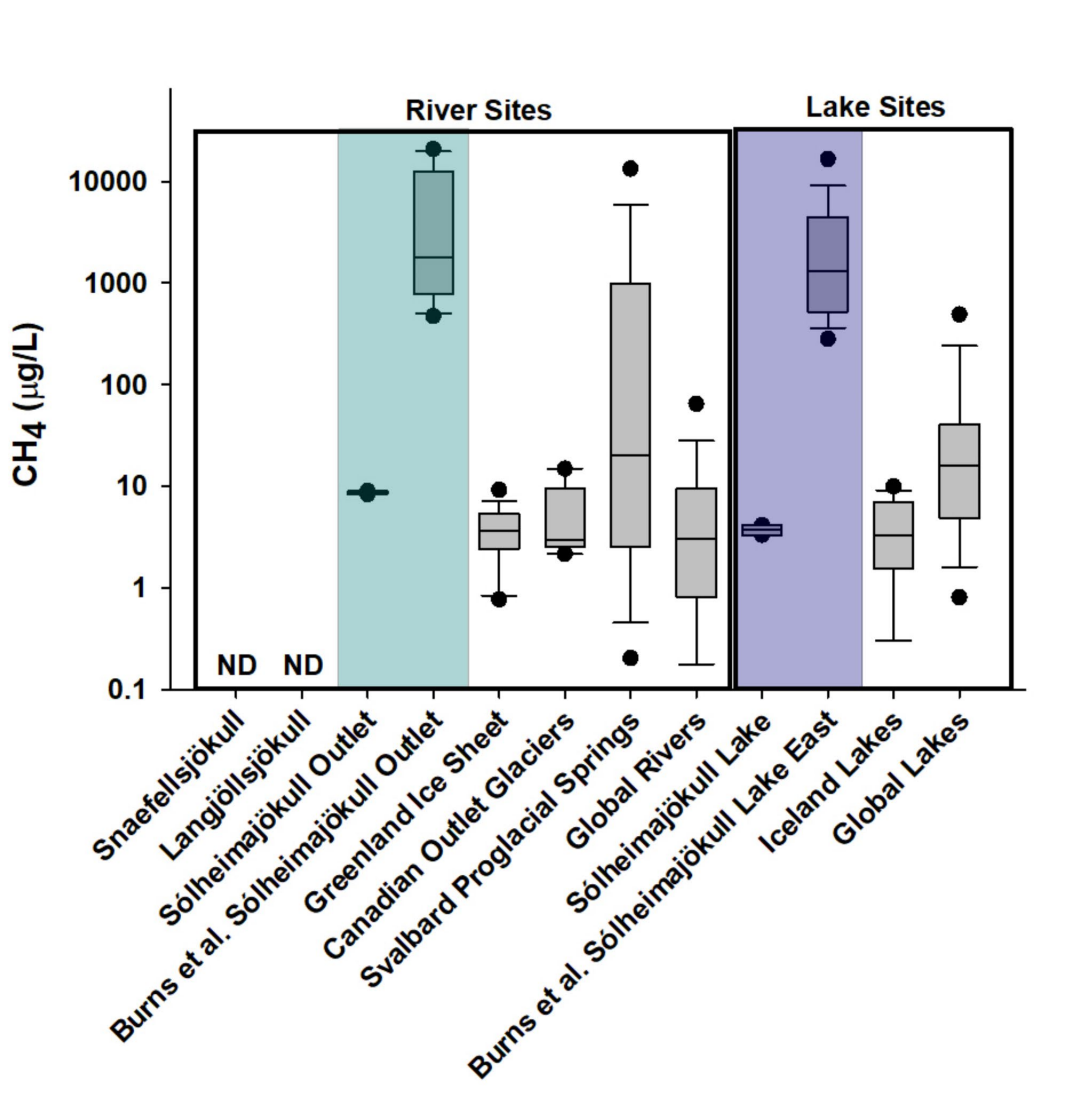
Methane concentrations in glacial, arctic, and global rivers (left) and lakes (right). River methane concentrations are from the three glacial rivers sampled in this study, a previous study by Burns et al.^7^ at one of this study’s sampling sites (river sites sampled repeatedly by different studies are highlighted in blue), a glacial river site from the Greenland Ice Sheet^8^, three outlet glaciers in Yukon, Canada^11^, groundwater springs from 78 land-terminating glaciers^10^ and global rivers^33^. Lake methane concentrations are from the paraglacial lake sampled in this study that was also studied by Burns et al.^7^ (highlighted in purple), a June survey of 14 Icelandic lakes (none of which were paraglacial)^31^, and global lakes^6^. Values from Burns et al.^7^ represent samples collected throughout late summer (on calendar day 185) in 2013, 2014, and 2017. Methane concentrations are displayed on a log scale. The boxes demarcate the 25th and 75th percentiles; the lines indicate the median concentrations; the whiskers extend to the largest value less than 1.5 times the inter quartile range, and data extending beyond this range are plotted as individual points. “ND” indicates non-detectible methane concentrations.

We measured strong net methane oxidation in paraglacial river sediments (2.97 µmol CH_4_ L^− 1^ d^− 1^) and weaker net methane production in the paraglacial lake sediments (0.12 µmol CH_4_ L^− 1^ d^− 1^). Glacial environments can produce and export high concentrations of methane; however, consumption in rivers and lakes on the glacial flood plain may significantly reduce emissions to the atmosphere (Fig. 2). In Sólheimajökull River, we measured net methane oxidation rates that were nearly an order of magnitude greater than the oxidation rate reported in Greenland marginal rivers (2.97 vs. 0.32 µmol CH_4_ L^− 1^ d^− 1^)^9^. To contextualize this net methane oxidation rate, we can estimate the amount of methane removed from the paraglacial stream via oxidation and compare areal rates of net oxidation to emission. Using winter and summer discharges (10–50 m^3^ s^−1^), river reach length (4 km), and width (20 m) reported in Burns^7^ and an average river depth of 1 m, and assuming hydraulic continuity to calculate water velocities, the residence time of water in the reach is between 27 min (summer discharge) and 133 min (winter discharge). If the net oxidation rate of 2.97 µmol CH_4_ L^− 1^ d^− 1^ that we measured is consistent throughout the reach, we estimate that the methane flux originating from glacial melt would be reduced by 11 to 53%, from an original river concentration of 0.53 μm down to 0.06–0.28 μm for summer and winter conditions respectively. When the same incubations are used to calculate an areal methane oxidation mass flux using the diameter of the bottle (41 mm) as surface sediment area, we measured net oxidation of 157.5 µmol CH_4_ m^− 2^ d^− 1^. These values are greater than the diffusive emission estimate of 63 µmol CH_4_ m^− 2^ d^− 1^, but they need to be interpreted with caution. Our bottle assays do not recreate the re-aeration that occurs due to river turbulence, and they only approximate the interaction between river water and sediments, but they do suggest that net methane oxidation has the capacity to significantly reduce atmospheric flux. If we apply the 2.97 µmol CH_4_ L^− 1^ d^− 1^ net oxidation rate we measured to the methane loading calculated via average aqueous methane concentrations in Burns et al.^7^ (48 Tonnes d ^− 1^), the oxidation is negligible, reducing river methane flux by < 0.001%. This is likely due to the much lower concentrations of methane measured during this study as compared to Burns et al.^7^ (Fig. 3), and the propensity for methane oxidation to be substrate limited^24^. This comparison highlights the importance of quantifying methane concentration, emission, and oxidation in tandem (rather than comparing different rate estimates made at different times as was done to rule out oxidation in LaMarche-Gagnon et al.^8^).

Globally, lakes are consistently net sources of methane to the atmosphere^6^, and reasoning follows that paraglacial lakes are a potential source of methane on the glacial landscape continuum. We report low levels of net methane production in the lake surface sediments relative to other lake ecosystems where methane production in sediments is commonly observed (e.g. Bastviken et al.^25^). In a study of glacial lakes of the Tibetan Plateau, Yan et al.^26^ also found lakes to be net sources of methane to the atmosphere with rates ranging from 0.07 ± 0.05 to 66 ± 13 mg m^− 2^ d^− 1^. Methane produced in proglacial lakes may be conflated with glacially-produced methane when measured in downstream riverine sites. Groundwater springs originating from 78 land-terminating glaciers in Svalbard were supersaturated in methane up to 600,000 times greater than atmospheric equilibration^10^. In Svalbard, this thermogenic methane traveled along subglacial groundwater flow paths and was brought to the surface with proglacial groundwater springs. If these springs enter a glacial lake or riverine system, the methane may be subject to microbial oxidation. The extremely low rates of net methane production estimated in the paraglacial lake sediments we sampled (1.9 µg CH_4_ m^− 2^ d^− 1^ ) relative to CH_4_ emissions from a global lake dataset (lowest reported value of 320 µg CH_4_ m^− 2^ d^− 1^)^6^ suggest that these systems may not be as strong a source as other lake types in the global methane budget. Similarly, Yan et al.^26^ reported low diffusive flux in glacial lakes with high methane emission rates attributed to ebullition. Yan et al.^26^ also highlighted the importance of groundwater input, substrate limitation and oxidation in highly variable lake methane flux in this region. Lake methanotrophy can mitigate a significant fraction of methane emissions in some systems, and is particularly efficient at doing so under oligotrophic conditions^27^. For example, in sub-arctic thermokarst lakes, oxidation reduced atmospheric effluxes by anywhere between 10 and 60%^28^ and similar proportional reductions in methane emission have been reported in wetland and temperate lake environments^18,20,29^.

### Spatial and temporal variability in methane concentrations complicate our understanding of methane oxidation in glacial systems

Counter to previous work in glacial systems^7,8^, methane concentrations during high melt were not universally elevated. Only one of the three glacial riverine systems (Sólheimajökull) had measurable methane, with values ranging from 8.3 to 8.9 µg L^− 1^ (Fig. 3). Our riverine values were in line with other glacial sites^8^ and global rivers^30^, suggesting that methane concentrations in glacial rivers may not differ significantly from other non-glacial riverine sites that span equatorial to polar latitudes and vary in size, land use, and biome. Methane concentrations in the paraglacial lake were similar to concentrations in lakes across southern and western Iceland (ranging from 0.0 to 9.9 µg L^− 1^^31^), although none of the lakes included in the Icelandic lake average were paraglacial (Fig. 3). All Icelandic values were considerably lower than the average methane concentration observed in fifteen Greenlandic lakes (40.48 µg L^− 1^), where methane concentrations increased with increasing distance from the ice sheet^32^. Overall, these lines of evidence suggest that paraglacial lakes are not a disproportionately large source of methane relative to other lake types.

In addition to spatial variability, methane delivery from glacial systems can be temporally variable. For example, lake and riverine methane concentrations measured at Sólheimajökull in this study were two orders of magnitude less than previously measured at this same site during peak-melt by Burns et al.^7^ (indicated by purple and blue bars respectively in Fig. 3). The Sólheimajökull site was selected for oxidation experiments based on these previous reports of high methane flux from the glacier during the summer melt period^7^. We timed our sampling to match high melt, when hypoxic conditions in the glacial bed and along the subglacial drainage pathway likely facilitate production and transport of methane rather than methane oxidation^7^. Our universally low methane concentrations support Burns et al.^7^ suggestion that methane concentrations are highly variable across seasons and years. This runs counter to conclusions by Lamarche et al.^8^ that methane transport from Greenland subglacial environments remained consistent across different melt conditions.

On a single sampling day, surface methane concentrations at Sólheimajökull Lake ranged from 3.3 to 4.1 µg L^− 1^, which were approximately half the downstream riverine values in Sólheimajökull outlet. Over multiple sampling years and seasons, Burns et al.^7^ consistently found higher concentrations of methane in the river site than the lake. The river site in Burns et al.^7^ is further downstream of the paraglacial lake-river nexus in this study system and the higher concentrations may include additional melt entering the system including via subsurface flows. Groundwater flow had the highest methane concentrations measured by Burns et al.^7^. In the Svalbard archipelago, groundwater elevated in methane is reaching surface water via springs that form in the talik as glaciers retreat^10^. Thus, sampling only the river or the paraglacial lake would not capture the full amount of methane produced or consumed in the floodplain system. In our study, the lower methane concentrations in the lake as compared to the river may imply disconnected hydrology and variable amounts of methane oxidation. This may not be the case in all systems; we suggest the importance of reaching across these ecosystem boundaries when designing studies to capture methane emission in glacial landscapes (Fig. 2). Our sampling occurred in close proximity to the glacier, which provides confidence that the fraction of carbon originating from terrestrial material in the watershed (as opposed to the glacial bed) is minimal. However, as sampling occurs further downstream from glacial inputs, evasion, oxidation and additional inputs of older terrestrial carbon may all influence dissolved methane concentrations.

### Directions for future research

Additional research could help constrain the role of methane oxidation in glacial landscapes. Initial findings support a large role for oxidation in the receiving waters of glacial melt in mitigating methane emissions to the atmosphere. Here we suggest three key steps to standardize the study of methane in glacial landscapes: (1) avoid quantifying glacial emissions by comparing concentrations between source and receiving waters, (2) pair estimates of methanogenesis and methane oxidation in time and space, and (3) standardize rate assays for easier comparison across sites. Following these guidelines will improve our capacity to better constrain this emission source.

Previous estimates of glacial methane flux to the atmosphere have calculated emissions as the difference between methane concentrations in meltwater and methane concentrations further downstream^7^. Estimating methane emissions as a difference discounts the possibility that methane oxidation (rather than emission) is the primary driver of methane loss from a glacial system. While simplistic, the approach is employed in other aquatic ecosystems that experience a high influx of methane such as in tailwaters below dams^34,35^. Given the high emissions that result from this approach in glaciers^7^ and given the high rates of net methane oxidation we report, we urge research across the glacial floodplain to measure methane emissions at the air water interface to better constrain atmospheric fluxes. In systems where watershed methane inputs are important (e.g. from terrestrial carbon and/or in situ methanogenesis in the river), the technique may also underestimate atmospheric losses by failing to account for additional methane inputs.

Methane flux estimates calculated with methanogenesis and methane oxidation rates collected at different times and locations should be used with caution. For example, previous efforts to constrain glacier-related methane emission have applied subglacial methane oxidation rates to the glacial river^8^. Based on our estimates, subglacial oxidation could underestimate the role of oxidation in downstream environments by an order of magnitude. More broadly, methanogenesis and methane oxidation are highly variable in space and time. Methane oxidizers are frequently substrate limited^24^ and upregulation of methane oxidizing genes and community members may lag behind pulses of methane. Communities of methane oxidizing microbes consist of bacteria and archaea and the slow growth of archaea may further slow ecosystem response to high methane concentrations.

Finally, reporting ancillary information from field and laboratory assays can facilitate cross-study comparisons. Given the redox sensitivity of both methane production and oxidation, information about the length of bottle assays (and the degree of oxygen depletion) is important. Differences in oxidation rate methods and in unit reporting also make rate comparisons across systems difficult, highlighting the value of identifying more broadly accepted and applied methods in this field. This is further complicated by inconsistencies in units (mg per kg of sediment versus mg per liter of water), which are required across studies to draw conclusions at larger spatial scales.

As the climate continues to warm and glacial melt increases across many polar landscapes, it is important to better constrain the potential impact on the global methane budget. Although four recent studies^7,8,10,11^ suggest large amounts of methane are being liberated from glacial landscapes, our study suggests that this is not characteristic of all glacial melt and that a significant portion of the methane leaving the glacier may be oxidized in glacial rivers before reaching the atmosphere. Rough upscaling of glacial methane emissions based on recent studies results in order of magnitude differences. This large uncertainty in upscaling emissions from glacial melt systems could be reduced by collecting measurements from more systems and across more timescales. In particular, incorporating methane oxidation rates from receiving melt rivers into upscaling calculations may help constrain emissions. In glacial melt systems with high levels of methane, studying the balance between methanogenesis and methanotrophy in receiving waters is critical to understand the role of atmospheric-water methane fluxes from paraglacial landscapes. A cross-system perspective is needed to fully understand the biogeochemical linkages that control methane evolution, transport and consumption across glacial environments.

## Methods

A total of four sites were sampled from three different Icelandic glaciers: Langjökull, Snæfellsjökull, and Sólheimajökull (Fig. 1). Sampling sites were all within 50 m of the glacier, sometimes adjacent to the ice (Fig. 2). The close proximity of sampling sites to the glacier is meant to best capture methane concentrations in glacier melt, with one study estimating that about 20% of exported methane reaches the atmosphere within 2 km of the ice sheet margin^8^. It also reduces the chance that other watershed methane carbon sources (such as from terrestrial carbon) are contributing to the methane concentrations we report. At Sólheimajökull, there was a paraglacial lake with a river (Jökulsá á Sólheimasandi) outlet, both of which were sampled independently.

Glacial runoff was collected as close to the base of each glacier as possible between June 19 and June 21 of 2019. Sampling in mid-June represents the period of high melt for these sites observed in previous studies^7^. Most of the glacial river water sampled in this study was turbid, with glacier flour suspended within the water collected close to the base of the glacier.

At each site, barometric pressure was measured with a Extech SD700 barometer, air and water temperature were measured with a Reference Thermapen (0.01 °C resolution). Two 70mL Wheaton vials, rinsed in triplicate, were flushed and over-filled using a triple-rinsed 100mL syringe and PVC vacuum tubing to eliminate bubbles from samples. One drop of super-saturated ZnCl_2_ solution was added to each vial before sealing with an aluminum crimp top. A 20mL ultra pure helium headspace was introduced in each vial upon returning to the laboratory, which was allowed to equilibrate with the water for 24 h before a 12mL gas sample was drawn from the headspace and injected into an exetainer (Fig. 3). Two exetainers from each site were used to measure methane concentrations via gas chromatography.

We also estimated the potential role of methane oxidation during the glacial melt season in waters draining Sólheimajökull Glacier. This target site was selected based on previous reports of high methane flux from the glacier during the summer melt period^7^. To quantify the potential role of oxidation, we incubated sediment and overlying water from both the paraglacial lake and river to measure the net accumulation (production) or depletion (oxidation) of methane over a 24-hour period in-situ. We then compare these net rates with the atmospheric diffusive emission rates estimated from ambient surface water concentrations.

Methane assays were conducted in the paraglacial lake and outflowing river of Sólheimajökull in order to calculate net methane flux at the sediment-water interface. Surface sediments from the lake and river were added to vials prior to adding lake or river water. The top 5 cm of sediment was collected from the edge of each site using a gravity sediment corer. The sediment was then homogenized and 5mL of the slurry was added to seven vials at each site (lake and river). After adding sediment, the vials were filled with water from each respective location using a triple-rinsed 100mL syringe and flexible Nalgene PVC tube. Three vials were treated with three drops of ZnCl_2_ to measure initial methane concentration. Four vials, filled with sediment and water from each sampling location, were sealed. These four vials were attached to an anchor and incubated in-situ (either lake or river) for 21 h (river) or 22 h (lake). Although samples were submerged at deployment, water levels fell over the incubation period and were exposed at retrieval. As a result, the sealed vials may have experienced a maximum air temperature (8.2 °C) as compared with the water temperature (0.9 to 1.0 °C) for a portion of the incubation. Even the maximum air temperature for this incubation (8.2 °C) was lower than some glacial river sites sampled as a part of this study (maximum water temperature of 12.4 °C across glacial drainage sites). The incubation period was chosen to maintain relatively oxygenated conditions within the bottle, to best mimic field conditions at the sediment water interface.

Upon retrieval, 2mL ZnCl_2_ were added to all vials to halt microbial activity. A 20mL helium headspace was added to the vials upon returning to the laboratory and allowed to equilibrate with the water and sediment for 24 h before a 12mL gas sample was drawn from the headspace and injected into an exetainer. A Yellow Springs Instruments 550 A in-situ sensor was used to measure dissolved oxygen in one vial from each site to measure oxygen consumption over the incubation period. Incubations remained aerobic, with final oxygen concentrations ranging from 16 to 17 mg/L. Methane concentrations were quantified using a gas chromatograph with a flame ionization detector and methanizer. Net methane production or consumption was calculated as the difference between the initial and final methane concentrations. A per liter rate was calculated based on the volume of water in the incubation bottles (70 mL), an areal rate was calculated based on the surface area of sediment in each bottle (1320 mm^2^), and a rate per mg of sediment was calculated based on the volume (5 mL) and bulk density (0.70–0.96 g mL^− 1^) of sediment in each bottle.

We estimated atmospheric methane emissions from Sólheimajökull River and Sólheimajökull Lake as a function of gas transfer and the relative supersaturation of methane in the surface waters:

$$F=k (Cw-Ceq).$$

where F is the areal flux of methane (mg CH_4_ m^− 2^ d^− 1^), Cw is the concentration of methane measured in the water (mg L^− 1^), Ceq is the concentration of surface water methane at equilibrium with the atmosphere (mg L^− 1^), and k is the gas transfer (m d^− 1^) for CH_4_ at the relevant temperature.

Gas transfer (k) was estimated by first modeling the gas transfer of CO_2_ at 20 degrees C (k_600_) based on published relationships to morphometric (Sólheimajökull River) and wind-related (Sólheimajökull Lake) variables. To estimate k600 in Sólheimajökull River, we first calculated energy dissipation according to Moog and Jirka^36^ and Raymond et al.^37^:

$$eD = gSv,$$

where *g* is the gravitational acceleration, *S* is the channel slope, and *v* is the velocity of the water. S and v were set at 0.01 and 4 m s^− 1^ respectively based on Lawler^38^. eD was then used to estimate k_600_ based on the high energy dissipation equation in Ulseth et al.^39^:

$$ln[k600]=6.43 + 1.18\times ln[eD]$$

k_600_ in Sólheimajökull Lake was estimated based on published relationships between k_600_ and wind speed. Wind conditions at the lake were approximated based on weather data from the nearby town of Ásólfsskáli, South, IS (63.6°N, 19.8°W) accessed in Climate Reanalyzer (where half of summer days had wind speeds above 3.7 m s^− 1^). We thus estimated k_600_ based on a wind speed > = 3.7 m s^− 1^ 10 m above the lake as 0.27 m d^− 1^ (Eq. 6 in Crusius and Wanninkhof^40^).

To relate k_600_ back to the k for CH_4_ at ambient temperature (kx), we calculated the Schmidt number (Scx) based on Wanninkhof^41^ and then used the established relationships between Schmidt numbers, wind speed, and k^35^:

$$\frac{k600}{kx}=\left(\frac{600}{Scx}\right)^{n}$$

where the exponent n was set to −½ for the turbulent river environment^42^ and for the windy conditions often present at the lake site^40^.

We compare per area rates of net methane oxidation measured via bottle assays with the atmospheric emission of methane estimated via thin boundary techniques. Per volume rates of oxidation measured in the bottles were compared to the residence time of the methane in the same 4 km reach as discussed by Burns et al.^7^, assuming both summer and winter discharge scenarios (50 and 10 m^3^ s^− 1^ respectively), 20 m river width, and 1 m river depth.

Global upscaling was done by first applying the glacial CH_4_ fluxes reported by Lamarche et al.^8^ and Burns et al.^7^ to the surface areas of the respective glaciers to determine a per area flux. We used a surface area of 20 km^2^ for Leverett Glacier^43^, 6,268 km^2^ for the Svalbard archipelago glaciers, and 9 km^2^ for Sólheimajökull^44^. We used methane fluxes of 6.3 and 2460 Mg CH_4_ yr^− 1^ from the Leverett and Sólheimajökull Glacier respectively. This resulted in 0.315 Mg CH_4_ km^− 2^ yr^− 1^ from the Leverett Glacier, 0.36 Mg CH_4_ km^− 2^ yr^− 1^ from the glacier of the Svalbard archipelago, and 273 Mg CH_4_ km^− 2^ yr^− 1^ from Sólheimajökull Glacier. We then upscaled these areal rates assuming a global glacier coverage^45^ of 680,000 km^2^.

## Data Availability

Methane measurements, including data from net methane oxidation assays, are publicly available at 10.5281/zenodo.13194967. The Strock et al. (2024) dataset also contains methane concentration data from a synthesis of relevant aquatic ecosystems, used to compare against the original measurements collected.
